# *Salvia miltiorrhiza*-Containing Chinese Herbal Medicine Combined With GnRH Agonist for Postoperative Treatment of Endometriosis: A Systematic Review and meta-Analysis

**DOI:** 10.3389/fphar.2022.831850

**Published:** 2022-02-16

**Authors:** Qiang Gao, Lei Shen, Bei Jiang, Yi-feng Luan, Li-na Lin, Fan-ci Meng, Chao-ying Wang, Hui-fang Cong

**Affiliations:** ^1^ Graduate School, Heilongjiang University of Traditional Chinese Medicine, Harbin, China; ^2^ Department of Traditional Chinese Medicine, Aerospace Center Hospital, Beijing, China; ^3^ The First Department of Gynecology, The Second Affiliated Hospital of Heilongjiang University of Traditional Chinese Medicine, Harbin, China

**Keywords:** *Salvia miltiorrhiza*, GnRH-a, Chinese herbal medicine, endometriosis, systematic review, meta-analysis

## Abstract

**Background:** Endometriosis is an estrogen-dependent gynecological inflammatory condition that may lead to infertility and recurrent pelvic pain. The purpose of this research was to determine the efficacy and safety of *Salvia miltiorrhiza*-containing Chinese herbal medicine (CHM) combined with gonadotropin-releasing hormone agonist (GnRH-a) for postoperative endometriosis management.

**Methods:**Eight databases were systematically searched before October 2021, including PubMed, Embase, Cochrane Library, Scopus, Web of Sceince, CNKI, VIP, and Wanfang. Finally, all randomized controlled studies comparing *Salvia miltiorrhiza*-containing CHM paired with GnRH-a to GnRH-a alone for postoperative endometriosis management were included.

**Results:** A total of 10 trials involving 836 patients were reported and analyzed. Compared with the control group, the *Salvia miltiorrhiza*-containing CHM combined with GnRH-a group showed significant superiority in decreasing endometriosis recurrence (risk ratio [RR] = 0.26; 95% confidence intervals [CI]: 0.16–0.41) and increasing the pregnancy rate ([RR] = 1.96; 95% CI: 1.58–2.44). Similarly, the effect of the *Salvia miltiorrhiza*-containing CHM combined with GnRH-a on CA-125 serum levels was positive (standardized mean difference [SMD] = -0.79; 95% CI: −1.11 to −0.47). Furthermore, this group showed a significant reduction in adverse effects.

**Conclusion:** The results indicate that *Salvia miltiorrhiza*-containing CHM may be a viable choice for postoperative endometriosis therapy, with the potential to enhance pregnancy while decreasing recurrence and adverse effects.

## Introduction

Endometriosis is a gynecological disease characterized by the presence of endometrial epithelium and stroma outside the uterine cavity, which causes chronic pelvic pain, dysmenorrhea, deep dyspareunia, dysuria and infertility (Patzkowsky, 2021). Endometriosis affects between 5 and 10% of reproductive-age women worldwide. The true incidence of endometriosis may be higher due to factors such as undiagnosed and clinical misdiagnosis (Zondervan, et al., 2020; Taylor et al., 2021). Current therapy options mostly involve pharmacological and surgical treatments, and it is well known that laparoscopic surgery is regarded as the gold standard for endometriosis treatment (Sindan et al., 2021). Unfortunately, over 50% of women undergoing surgery require further surgery within 5 years (Saraswat et al., 2018; Saunders and Horne, 2021). GnRH-a is frequently used after surgery to eliminate microscopic lesions and prevent recurrence of endometriosis. It inhibits follicle development and ovulation by lowering FSH and LH secretion, which decreases the synthesis of estradiol and progesterone to suppress the progression of the lesion and treat endometriosis-related pain (Della Corte et al., 2020). Nonetheless, the long-term use of GnRH-a causes adverse effects such as vasomotor symptoms, insomnia, and bone density loss (Sauerbrun-Cutler and Alvero, 2019). Although add-back treatment is commonly used to alleviate side effects, its efficacy remains inadequate (Donnez and Dolmans, 2021).

Chinese herbal medicine (CHM) has long been used to treat various gynecological disorders, including endometriosis. The herb *Salvia miltiorrhiza* has been officially listed in Chinese Pharmacopoeia for treatment of menstrual disorder and blood circulation diseases and prevention of inflammation (Kuang et al., 2005). In addition, the use of *Salvia miltiorrhiza* in combination with Gui Zhi Fu Ling Wan formulation (Gynoclear™) is considered to reduce the severity and duration of aperiodic pelvic pain, dysmenorrhea, dyspareunia, and other endometriosis symptoms (Armour et al., 2021). Currently, known salvianolic compounds that have primarily pharmacological effects include tanshinone, salvianolic, rosmarinic, caffeic, protocatechuic and danshensu acids, which not only inhibit platelet aggregation and fibrosis, but also have anti-inflammatory, anti-oxidant effects, anti-cancer, and other pharmacological effects (Matkowski et al., 2008; Bostanci et al., 2021; Meresman et al., 2021). Tanshinone IIA, a pharmacologically active extract of *Salvia miltiorrhiza*, has been shown to control the renin-angiotensin system and diminish mechanical hyperalgesia in endometriosis pain (Chen and Gong, 2020). Furthermore, it inhibits the proliferation, migration, and invasion control of endometrial stromal cells, preventing lesions’ evolution (Luo et al., 2020).

Cancer antigen 125 (CA-125) is a glycoprotein, which is a well-established tumour marker of the ovarian epithelial cells. At present, CA-125 is considered to be a potential marker of endometriosis and has been widely tested in the clinical diagnosis of endometriosis. Although CA-125 has a relatively low sensitivity and specificity, its high level is related to the stage and clinical type of endometriosis and is more sensitive to stages III and IV of endometriosis (Kimber-Trojnar et al., 2021). It is recommended that concentrations of CA-125 be measured during the middle of the menstrual cycle and during the menstrual period. In particular, positive results for CA-125 in the middle of the menstrual cycle suggest a very high risk of endometriosis (Oliveira et al., 2017).

No previous systematic reviews or meta-analyses have been conducted to investigate the efficacy of *Salvia miltiorrhiza*-containing CHM in postoperative endometriosis patients. Therefore, this study aimed to evaluate the effect of *Salvia miltiorrhiza*-containing CHM combined with GnRH-a to reduce the risk of recurrence and adverse effects, and promote pregnancy in postoperative endometriosis patients.

## Materials and Methods

### Data Sources and Search Strategy

The search was conducted utilizing the following electronic databases to October 2021: PubMed, Embase, Cochrane Library, Scopus, Web of Sceince, China National Knowledge Infrastructure (CNKI), Journal Integration Platform (VIP) and Wanfang. Furthermore, to minimize publication bias, we manually evaluated the references of all selected studies and searched for related papers such as letters, research reports, research papers, conference proceedings, and abstracts. Medical subheadings (MeSH) words combined with free words were used for retrieval in the English library, and the search terms (endometriosis OR endometrioses OR endometrioma OR endometriomas) AND (Chinese Traditional Medicine OR Chinese herbal medicine) AND (GnRH agonist OR Gonadotropin-releasing hormone agonist OR GnRH-a) were used as keywords.

### Selection Criteria

#### Types of Studies

Included studies were human randomized controlled trials (RCTs) published in English or Chinese only. Non-RCTs, *in vitro* studies, and animal studies were removed. Reviews, case reports, abstracts, and repeated publications were also excluded.

### Types of Patients

Patients had a clear diagnosis of endometriosis, which was confirmed by pathological diagnosis of laparoscopic surgery or conservative surgery by laparotomy. Additionally, following surgery, all patients in the intervention and control groups were given GnRH-a therapy.

### Types of Interventions

Patients in the intervention groups received *Salvia miltiorrhiza*-containing CHM combined with GnRH-a as postoperative medical treatment, while patients in the control groups received GnRH-a alone therapy after surgery. *Salvia miltiorrhiza*-containing CHM was available as capsules, tablets, pills, and decoctions. Studies using Chinese nonherbal medicinal therapies such as acupuncture, external enema, cupping, or point application were excluded.

### Types of Outcome Measures

The primary outcomes were endometriosis recurrence rates. Secondary outcomes included pregnancy rate, CA-125 level in peripheral blood, and adverse events such as gastrointestinal reaction, vaginal bleeding, hot flashes and abnormal liver function.

### Data Extration

Two authors independently selected the articles based on inclusion and exclusion criteria. The following information was extracted using a standardized data collection form: first author, publication year, study design, sample sizes, intervention details, outcomes, and follow-up duration. When two authors disagreed, a third author joined the discussion to achieve an agreement.

### Quality Assessment

Two independent reviewers assessed the methodological quality of each trial using the Cochrane Handbook for Systematic Reviews of Interventions, which included items such as randomization process, deviations from intended interventions, missing outcome data, measurement of the outcome, selection of the reported result, and overall bias (Higgins et al., 2019).

### Statistical Analysis

The data analysis was performed with STATA software (version 16.0, Stata Corporation, College Station, TX, United States). Dichotomous variables were shown as risk ratio (RR) with 95% confidence intervals (CI), and continuous variables were presented as standardized mean difference (SMD) with 95%. Homogeneity across trails was evaluated using the *I* ^*2*^ statistics, and *I* ^*2*^ > 50% was assumed to have high heterogeneity. We applied a fixed-effect model to assess treatment effects. A *p*-value < 0.05 was considered statistically significant.

## Results

**FIGURE 1:**
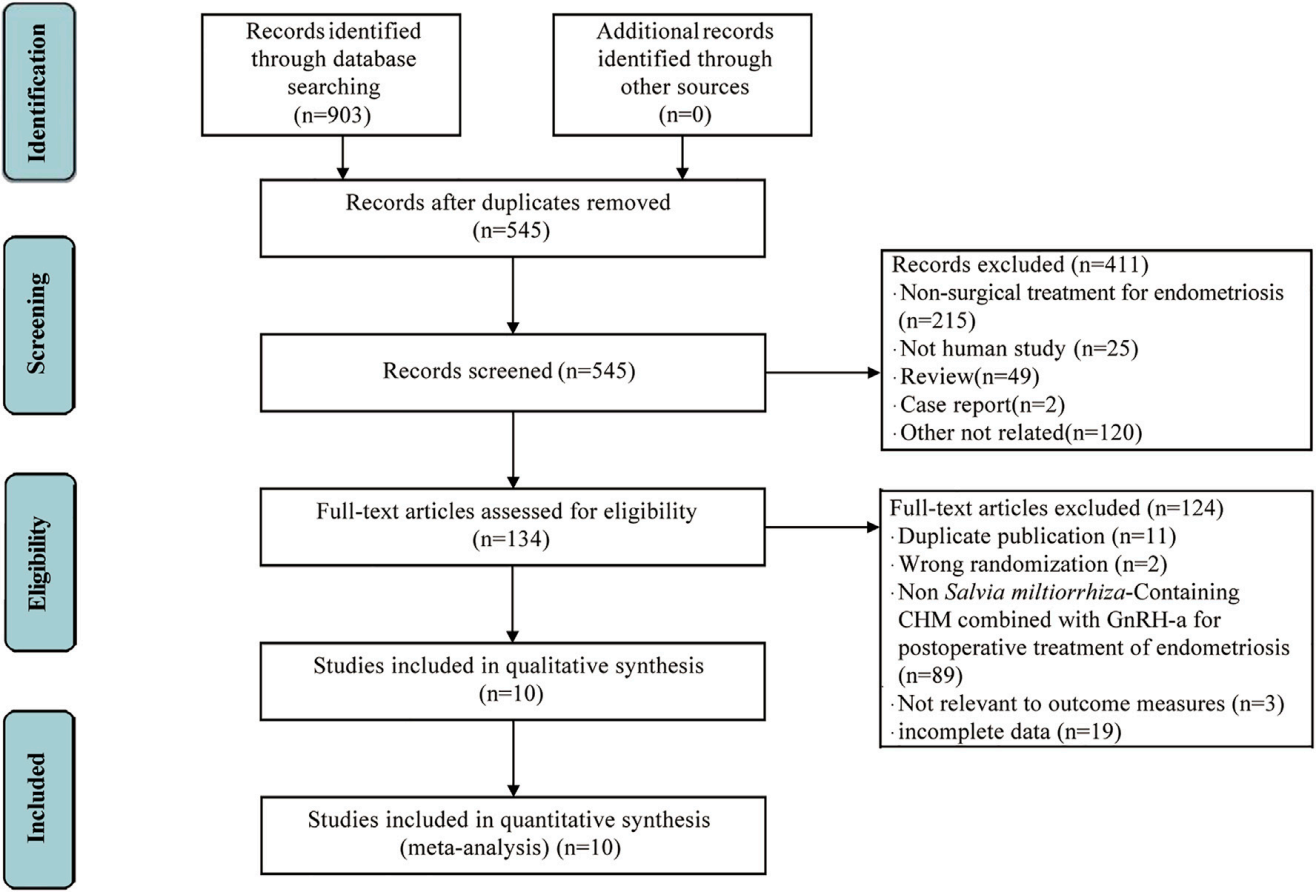
Flow chart of studies selection process.

The screening process is shown in Figure 1, 903 articles were selected by searching Pubmed (*n* = 11), Embase (*n* = 14), the Cochrane library (n = 9), Scopus (n = 2), Web of Sceince (*n* = 15), CNKI (*n* = 191), VIP (*n* = 241), and the Wangfang database (*n* = 420). A total of 358 articles were removed after deleting the duplicates. 411 articles were excluded after scanning the titles and abstracts. Following the full texts 134 studies reviewed, only 10 studies met the criteria were finally included in this systematic review and meta analysis.

### Study Characteristics

All included studies were conducted in China and published in Chinese between 2016 and 2021. Table 1 shows the main characteristics of the trails, which consisted of sample size, age, duration, and outcomes. 10 studies recruited a total of 836 postoperative patients with endometriosis, including 420 patients in *Salvia miltiorrhiza*-containing CHM combined with GnRH-a group and 416 patients in GnRH-a alone group.

**TABLE 1:** Characteristics of included studies.

Study	Sample size (TG/CG)	Age(years)	Intervention group	Control group	Duration	Follow-up duration	Outcomes
Chen (2019)	100 (50/50)	TG: 30.73 ± 4.93	Salvia-containing	Leuprolide Acetate	CHM: 21 days * 3 courses	12 months	①
		CG: 30.90 ± 4.75	CHM + Leuprolide Acetate		Leuprolide Acetate: Once every 28 days * 3 courses		
Du et al. (2018)	60 (30/30)	TG: 38.0 ± 1.5	Salvia-containing	Goserelin Acetate	CHM: 3 weeks * 6 courses	24 months	①②③④
		CG: 37.1 ± 1.5	CHM + Goserelin Acetate		Goserelin Acetate: Once every 4 weeks * 6 courses		
Hu (2016)	92(46/46)	TG: 34.1 ± 4.7	Salvia-containing	Triptorelin	CHM: 4 weeks * 6 courses	24 months	①②
		CG: 33.9 ± 4.9	CHM + Triptorelin		Triptorelin: Once every 4 weeks * 6 courses		
Hu and Kuang (2018)	90(45/45)	TG: 30.9 ± 5.5	Salvia-containing	Triptorelin Acetate	CHM: 14 days * 6 courses	6 months	②③④
		CG: 30.2 ± 6.1	CHM + Triptorelin Acetate		Triptorelin Acetate: Once every 28 days * 6courses		
Lei and Gao(2019)	104 (52/52)	TG: 28.71 ± 5.06	Salvia-containing	Leuprolide + Estradiol Valerate	CHM: 28 days * 6 courses	36 months	①②③④
		CG: 29.86 ± 4.83	CHM + Leuprolide + Estradiol Valerate		Leuprolide: Once every 28 days * 6 courses		
Liu et al. (2021)	100 (52/48)	TG: 30.24 ± 4.30	Salvia-containing	Leuprolide Acetate	CHM: 12 weeks	12 months	①②④
		CG: 29.74 ± 4.12	CHM + Leuprolide Acetate		Leuprolide Acetate: Once every 4 weeks * 3 courses		
Wu (2016)	60 (30/30)	TG: 35.6 ± 2.62	Salvia-containing	Triptorelin Acetate	CHM: 21 days * 3 courses	6 months	②③
		CG: 36.6 ± 2.49	CHM + Triptorelin Acetate		Triptorelin Acetate: Once every 28 days * 3 courses		
Zhang (2020)	98(49/49)	TG: 35.02 ± 2.13	Salvia-containing	Triptorelin Acetate	CHM: 3 weeks * 6 courses	12 months	①②③④
		CG: 34.56 ± 2.67	CHM + Triptorelin Acetate		Triptorelin Acetate: Once every 4 weeks * 6 courses		
Zhang and Wang (2019)	60 (30/30)	TG: 31.63 ± 5.12	Salvia-containing	Leuprolide Acetate	CHM: 21 days * 3 courses	12 months	①②③④
		CG: 30.17 ± 4.47	CHM + Leuprolide Acetate		Leuprolide Acetate: Every 28 days * 3 courses		
Zhang and Zhao (2017)	72 (36/36)	TG: 30.7 ± 4.6	Salvia-containing	Leuprolide Acetate	CHM: 21 days * 3 courses	12 months	②④
		CG: 31.9 ± 5.0	CHM + Leuprolide Acetate		Leuprolide Acetate: Once every 28 days * 3 courses		

CHM = chinese herbal medicine; ①pregnancy rate; ②recurrence rate; ③CA-125; ④adverse events.

### Quality Assessment

**FIGURE 2:**
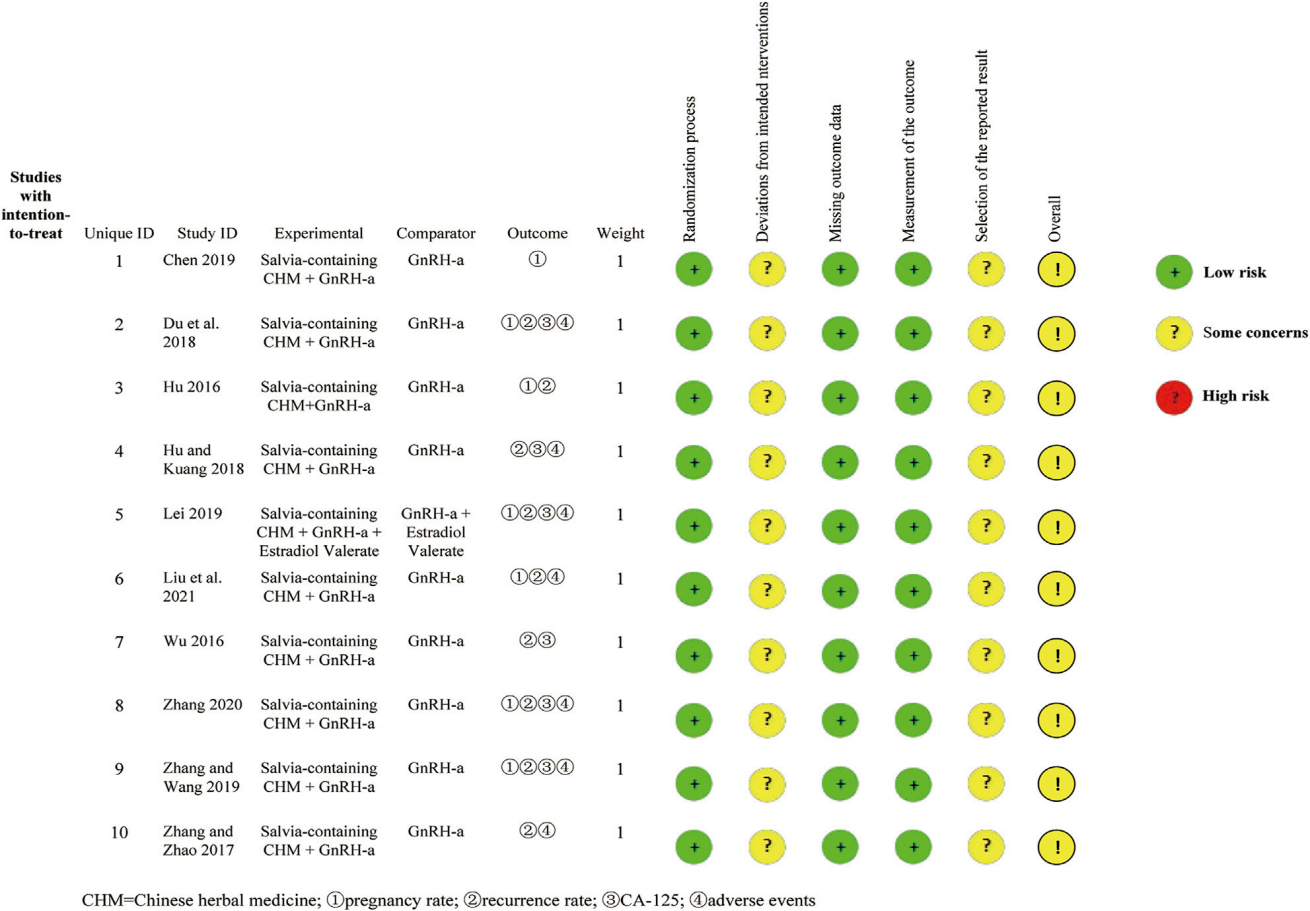
Risk of bias summary.

Figure 2 shows the methodological quality assessment of included studies. All studies were described as randomized, of which 7 studies used random tables. There were no statistically significant differences in baseline between the intervention and control groups across all enrolled studies. None of the included studies reported blind intervention for patients. There was no information that the intervention deviated from the intended intervention due to the experimental context. Outcome data was obtained for nearly all randomized groups of subjects. Although all studies were unclear on the blinding of outcome assessment, patients with endometriosis have objective evaluation indexes for recurrence, pregnancy and serum CA-125 level, and it was difficult to affect the outcomes evaluation. No information mentioned that the results analysed in accordance with published pre-specified analysis plan. Consistent outcome measures and data analysis methods were used for all included studies. Overall bias showed some concerns.

### Recurrence Rate

**FIGURE 3:**
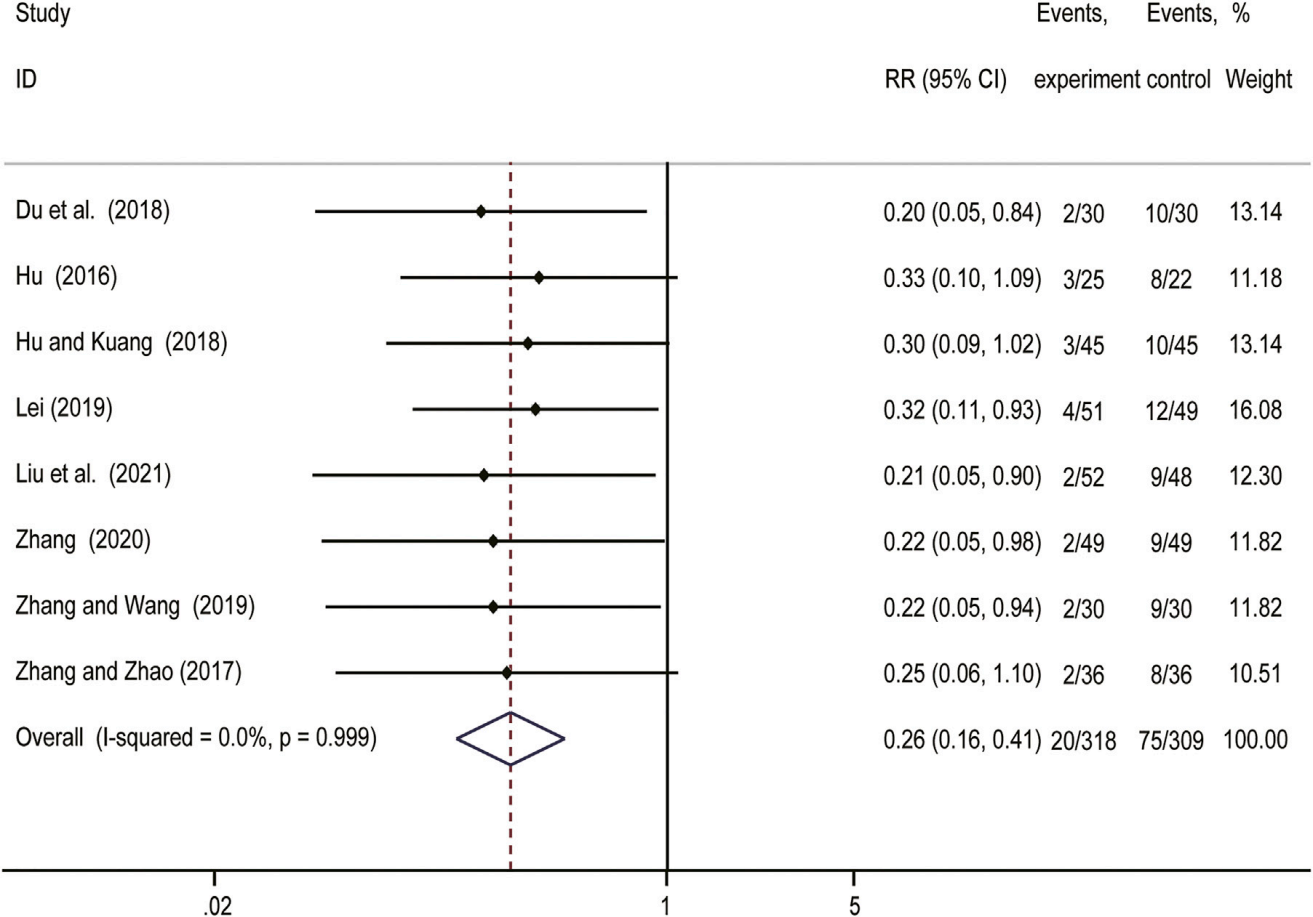
Forest plot showing comparision of recurrence rate comparing *Salvia*-containing CHM combined with GnRH-a to GnRH-a alone treatment.

As shown in Figure 3, a fixed effect model was used due to no obvious heterogeneity observed (*I* ^*2*^ = 0%, *p* = 0.999). The result (RR = 0.26; 95%CI: 0.16–0.41) indicated that *Salvia miltiorrhiza*-containing CHM plus GnRH-a was superior to GnRH-a alone in decreasing the recurrence rate.

### Pregnancy Rate and Serum Level of CA-125

**FIGURE 4:**
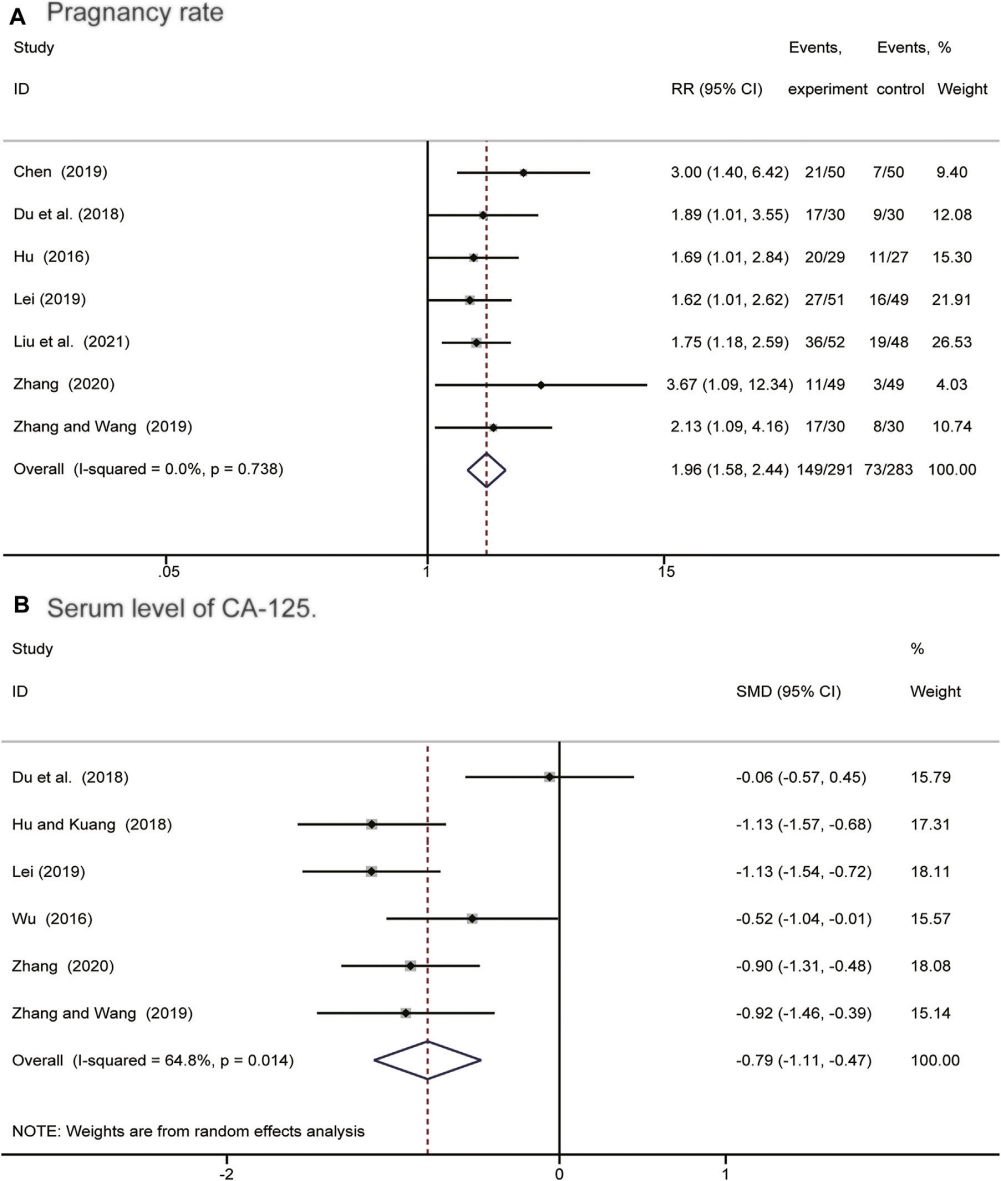
Forest plots showing comparision of pregnangcy rate **(A)** and serum level of CA-125 **(B)** comparing *Salvia*-containing CHM combined with GnRH-a to GnRH-a alone treatment.

As shown in Figure 4A, we applied a fixed effect model because no obvious heterogeneity was observed (*I* ^*2*^ = 0%, *p* = 0.738). The result (RR = 1.96; 95% CI: 1.58–2.44) showed that postoperative *Salvia miltiorrhiza*-containing CHM combined with GnRH-a therapy significantly increased the endometriosis pregnancy rate. Six studies compared the variation in serum CA-125 level between intervention and control groups. As depicted in Figure 4B, meta-analysis using a random model suggested that postoperatie *Salvia miltiorrhiza*-containing CHM combined with GnRH-a treatment remarkably reduced serum level of CA-125 (SMD = -0.79, 95% CI: −1.11 to −0.47) compared to GnRH-a alone.

### Adverse Events

**FIGURE 5:**
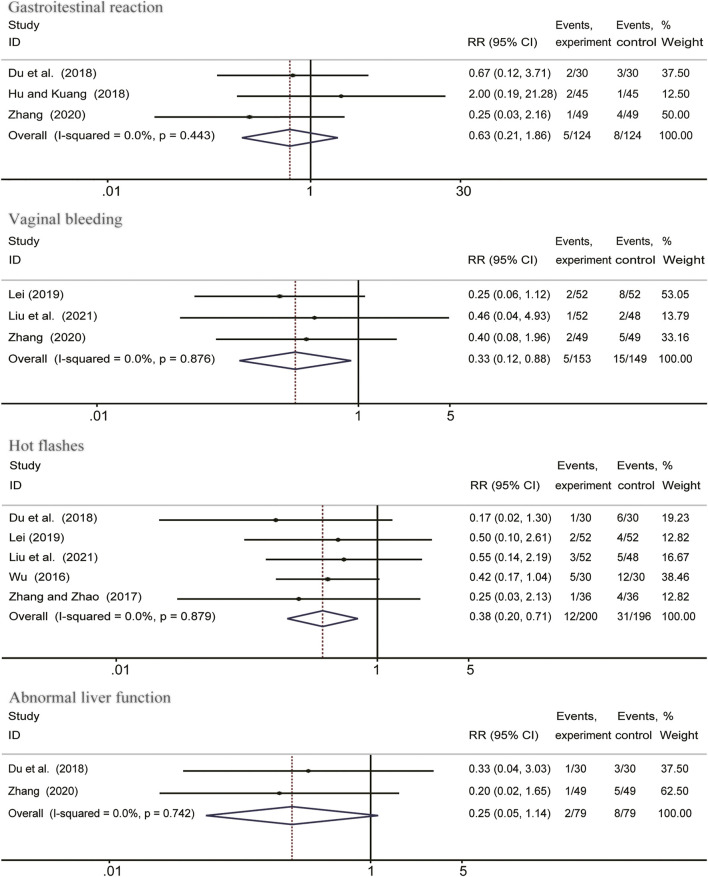
Forest plots showing comparision of adverse events comparing *Salvia*-containing CHM combined with GnRH-a to GnRH-a alone treatment.

Adverse events including gastrointestinal reactions, vaginal bleeding, hot flashes and abnormal liver function, were reported in 7 studies. As shown in Figure 5, meta-analysis indicated that the incidence of irregular vaginal bleeding (RR = 0.33; 95%CI: 0.12 to 0.88; *I* ^*2*^ = 0%, *p* = 0.876) and hot flashes (RR = 0.38; 95% CI: 0.20 to 0.71; I^2^ = 0%, *p* = 0.879) were lower in the *Salvia miltiorrhiza*-containing CHM combined with GnRH-a group than in the GnRH-a alone group. However, there were no significant differences on the risk of gastrointestinal reaction (RR = 0.63; 95% CI: 0.21 to 1.86; *I* ^*2*^ = 0%, *p* = 0.443) and abnormal liver function (RR = 0.25; 95% CI: 0.05 to 1.14; *I* ^*2*^ = 0%, *p* = 0.742).

### Subgroup Analyses and Sensitivity Analysis

**FIGURE 6:**
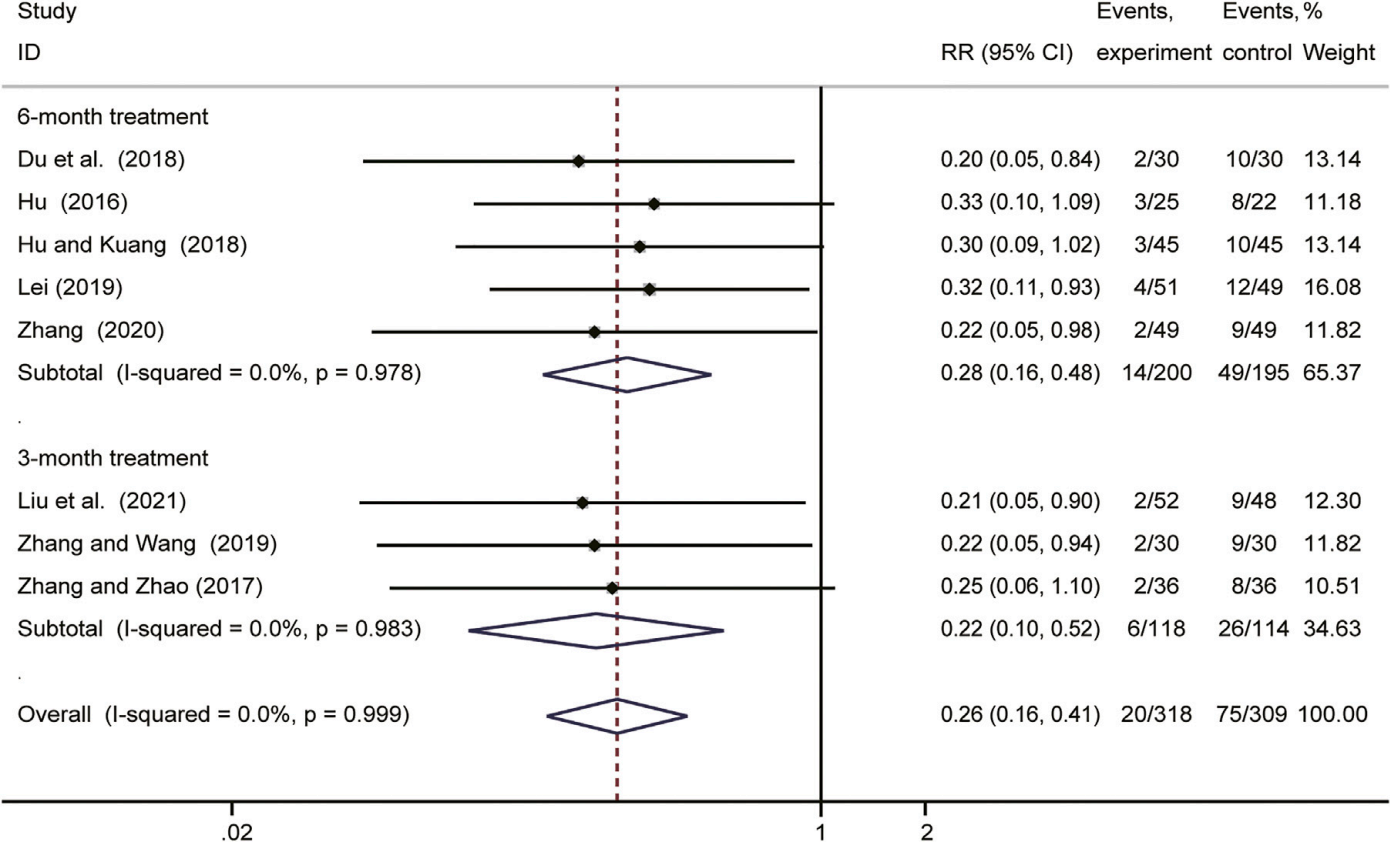
Forest plot of the effect of different durations of postoperative *Salvia*-containing CHM combined with GnRH-a treatment in preventing endometriosis recurrence.

According to the duration of therapy, we conducted the subgroup analysis on the recurrence rate in postoperative patients with endometriosis. The results revealed that the recurrence rate in patients received 6 months postoperative *Salvia miltiorrhiza*-containing CHM combined with GnRH-a treatment (RR = 0.28; 95% CI: 0.16–0.48) was much lower than those received 3 months treatment (RR = 0.22; 95% CI: 0.10–0.52) (Figure 6). The results of this meta-analysis can be considered stable since no significant changes were noted in the leave-one-out sensitivity analysis.

### Publication Bias

**FIGURE 7:**
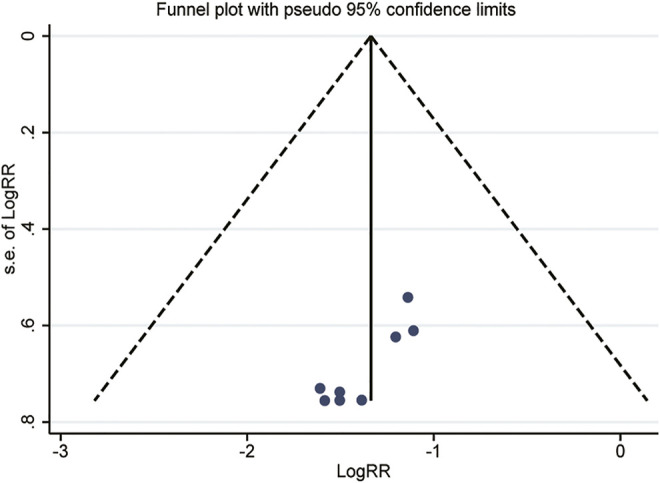
Funnel plot of the recurrence rate.

We used a series of approaches to investigate potential publication bias. Figure 7 presented that the funnel plot was generally symmetrical visually, and the result of Harbord’s test (*p* = 0.143) also confirmed it, which showed that publication bias was not obvious.

### Grade Evaluation of Evidence Quality

According to the GRADE standard (Guyatt et al., 2008), GRADE profiler 3. 6 was used to evaluate the evidence quality of each outcome. Outcome indexes were classified into four grades of high quality, medium quality, low quality and extremely low quality according to five aspects of research limitation, inconsistency, inaccuracy, indirectness and other biases. The Evidence Profifile with quality assessment and Summary of Findings were reported in Table 2.

**TABLE 2:** GRADE rating of the quality of each outcome.

Anticipated absolute effects* (95% CI)					
	Risk with	Risk with	Relative effect	№ of participants	Certainty of the evidence
Outcomes	[comparison]	[intervention]	(95% CI)	(studies)	(GRADE)
Recurrence rate	243 per 1,000	63 per 1,000 (39–100)	RR 0.26(0.16–0.41)	627 (8 RCTs)	⨁⨁⨁⨁
					High
Pregnancy rate	258 per 1,000	506 per 1,000 (408–629)	RR 1.96 (1.58–2.44)	574(7 RCTs)	⨁⨁⨁◯
					Moderate
serum level of CA-125	—	SMD 0.79 SD lower (1.11 lower to 0.47 lower)	—	472 (6 RCTs)	⨁⨁⨁◯
					Moderate
Gastrointestinal reactions	65 per 1,000	41 per 1,000< (14–120)	RR 0.63 (0.21–1.86)	248 (3 RCTs)	⨁⨁ ◯ ◯
					Low
Vaginal bleeding	101 per 1,000	33 per 1,000 (12–89)	RR 0.33(0.12–0.88)	302< (3 RCTs)	⨁⨁⨁◯
					Moderate
Hot flashes	158 per 1,000	60 per 1,000(32–112)	RR 0.38 (0.20–0.71)	396 (5 RCTs)	⨁⨁⨁◯
					Moderate
Abnormal liver function	101 per 1,000	25 per 1,000 (5–115)	RR 0.25 (0.05–1.14)	158(2 RCTs)	⨁⨁ ◯ ◯
					Low
Recurrence rate(3-month treatment)	228 per 1,000	50 per 1,000 (23–119)	RR 0.22 (0.10–0.52)	232 (3 RCTs)	⨁⨁⨁◯
					Moderate
Recurrence rate(6-month treatment)	251 per 1,000	70 per 1,000 (40–121)	RR 0.28 (0.16–0.48)	395 (5 RCTs)	⨁⨁⨁◯
					Moderate

*The risk in the intervention group (and its 95% confidence interval) is based on the assumed risk in the comparison group and the relative effect of the intervention (and its 95% CI). CI: confidence interval; RR: risk ratio; SMD: standardised mean difference.

## Discussion

Endometriosis is a common disease in women of reproductive age. Because of the mild symptoms at the beginning of the disease, patients do not seek immediate medical attention until the endometriosis has developed to a severe degree, increasing treatment difficulties. Some studies in recent years have comfirmed the clinical effects of *Salvia miltiorrhiza*-containing CHM for angina, myocardial infarction, hepatitis, and dysmenorrhea, as well as its low toxic side effects (Wu et al., 2012; Liao et al., 2019). Still, there is no systematic evidence to show whether *Salvia miltiorrhiza*-containing CHM is suitable for endometriosis. As far as we know, this is the first systematic review and meta-analysis of RCTs on the efficacy of *Salvia miltiorrhiza*-containing CHM in the treatment of endometriosis.

The meta-analysis’s main results revealed that, compared to GnRH-a therapy alone, *Salvia miltiorrhiza*-containing CHM + GnRH-a might reduce the recurrence rate of postoperative endometriosis patients. In addition, using *Salvia miltiorrhiza*-containing CHM as an adjuvant treatment to GnRH-a significantly increased pregnancy rates while decreasing peripheral blood CA-125 levels and side events. We systematically evaluated the results of 10 RCTs comprising 836 patients (420 in *Salvia miltiorrhiza*-containing CHM combined with GnRH-a group and 416 in the GnRH-a alone group).

Due to the high recurrence rate of endometriosis after surgery, inhibition of endogenous estrogen production is very important for the treatment of endometriosis (Bedaiwy et al., 2017). The combination of surgery and post-surgical medical treatment with GnRH-a is the most commonly used therapy for mediate and severe endometriosis, because GnRH-a has been proven to be effective in removing microscopic lesions and suppressing the secretion of FSH, LH and estradiol (Donnez et al., 2002). The CA125 is usually described as a clinical marker for the diagnosis of endometriosis (Kimber-Trojnar et al., 2021). In the current meta-analysis, *Salvia miltiorrhiza*-containing CHM combined with GnRH-a significantly decreased the serum level of CA125 in postoperative patients with endometriosis. Moreover, the preclinical study has proved its effect of treating endometriosis by significantly reducing rat serum CA-125 levels and IL-18 in peritoneal fluid, and increasing the level of IL-13 in peritoneal fluid (Zhou et al., 2012).

However, the pharmacological mechanism of GnRH-a in the treatment of endometriosis is down-regulation of the pituitary and suppression of gonadotropin release to sustain the hypo-estrogenic state, which causes mimic postmenopausal symptoms included irregular vaginal bleeding, hot flashes, headache (Rahmawati et al., 2019; Dai et al., 2021). Although hormone replacement “add-back” therapy is frequently prescribed as an adjunct therapy to GnRH-a to prevent menopausal side effects, long-term use of GnRH-a may lead to increasing the risk of osteoporosis (Alshehre et al., 2020). Given the above factors, the safety and effectiveness of using GnRH-a beyond 6 months are still controversial, which undoubtedly limit the clinical efficacy of GnRH-a (Bedaiwy and Casper, 2006; Hornstein, 2017). Therefore, it is necessary to seek complementary and alternative therapies to reduce the side effects of GnRH-a in the treatment of endometriosis. According to the results of this meta-analysis, *Salvia miltiorrhiza*-containing CHM combined with GnRH-a reduced the risk of vaginal bleeding and hot flashes caused by GnRH-a and did not significantly increase the incidence of gastrointestinal reactions and abnormal liver function. Furthermore, when taken the duration of *Salvia miltiorrhiza*-containing CHM treatment into account, we found that 6 months treatment with *Salvia miltiorrhiza*-containing CHM could better reduce the recurrence rate of endometriosis. These results indicated that the combination of *Salvia miltiorrhiza*-containing CHM and GnRH-a could improve the therapeutic effects. We believe that these benefits of *Salvia miltiorrhiza*-containing CHM make it a good candidate for the treatment of endometriosis.

There are several limitations in this study. First, some enrolled RCTs did not explicitly report a randomized approach, with only seven studies reported using random number tables and the remaining three mentioned randomization without supplying further details. None of the included studies reported implementation of a blinding method and using a placebo in the control group. These methodological flaws might generate bias, so that our results should be explained very carefully. Second, most studies lacked sample size evaluation, which leads to the low accuracy of most included small sample size studies. Third, the forms of *Salvia miltiorrhiza*-containing CHM were not unified, which might cause potential bias. Fourth, the missing stages of endometriosis reported in most trials might affect the pooling outcome for recurrence of endometriosis. Finally, all the included studies did not provide the approval results of the ethics committee, so we should pay attention to ethical issues and protect patients’ reasonable rights.

In conclusion, the findings of this meta-analysis indicated that *Salvia miltiorrhiza*-containing CHM might be used as a supplemental therapy for postoperative endometriosis treatment. *Salvia miltiorrhiza*-containing CHM appears to be a potential medication for improving clinical effectiveness and lowering GnRH-a side effects. However, due to the low quality of most of the included studies, further large-scale and high-quality, rigorous RCTs will be required in the future to confirm these results.

## Data Availability

The original contributions presented in the study are included in the article/Supplementary Material, further inquiries can be directed to the corresponding authors.
