# Supplementary material for: Salvia miltiorrhiza-Containing Chinese Herbal Medicine Combined With GnRH Agonist for Postoperative Treatment of Endometriosis: A Systematic Review and meta-Analysis
Source: Front Pharmacol. 2022 Feb 16;13:831850. doi: 10.3389/fphar.2022.831850 (PMC8889030; doi:10.3389/fphar.2022.831850)
Supplement: Supplementary file 1 [file Table1.docx]

| **Supplementary material Table 1，Described in manuscript lines 266-267.** | | | |
| --- | --- | --- | --- |
| **Section and Topic** | **Item #** | **Checklist item** | **Location where item is reported** |
| **TITLE** | | |  |
| Title | 1 | The report is identified as a systematic review. | 1 |
| **ABSTRACT** | | |  |
| Abstract | 2 | The structured abstract contains Background, Methods, Results and Conclusion. | 1 |
| **INTRODUCTION** | | |  |
| Rationale | 3 | Described in the introduction. | 1-2 |
| Objectives | 4 | this study aimed to evaluate the effect of Salvia-containing CHM combined with GnRH-a on the reduction of recurrence rate and side effects and improvement of pregnancy rate in postoperative patients with endometriosis. | 2 |
| **METHODS** | | |  |
| Eligibility criteria | 5 | Included studies were randomized controlled trials (RCTs) in humans only and published in English or Chinese. Non-RCTs, in vitro studies, and animal studies were removed. Reviews, case reports, abstracts, and repeated publications were also excluded. | 3 |
| Information sources | 6 | The search was conducted using PubMed, Embase, Cochrane Library, Scopus, Web of Sceince, China National Knowledge Infrastructure (CNKI), Journal Integration Platform (VIP) and Wanfang electronic databases before October 2021. | 2 |
| Search strategy | 7 | Medical subheadings (MeSH) words combined with free words were used for retrieval in the English libraries, while the search terms (endometriosis OR endometrioses OR endometrioma OR endometriomas) AND (Chinese Traditional Medicine OR Chinese herbal medicine) AND (GnRH agonist OR Gonadotropin-releasing hormone agonist OR GnRH-a) were used as keywords. | 2-3 |
| Selection process | 8 | Described in the data extration | 3 |
| Data collection process | 9 | Described in the data extration | 3 |
| Data items | 10a | Described in the data extration | 3 |
|  | 10b | Described in the data extration | 3 |
| Study risk of bias assessment | 11 | Described in the Quality Assessment | 3 |
| Effect measures | 12 | Statistical Analysis  Risk ratio or standardized mean difference was used in the synthesis or presentation of results. | 4 |
| Synthesis methods | 13a | Described in the statistical analysis | 4 |
|  | 13b | Described in the statistical analysis | 4 |
|  | 13c | Described in the statistical analysis | 4 |
|  | 13d | Described in the statistical analysis | 4 |
|  | 13e | Described in the statistical analysis | 4 |
|  | 13f | Described in the statistical analysis | 4 |
| Reporting bias assessment | 14 | Homogeneity across trails was evaluated using the I^2^ statistics. | 4 |
| Certainty assessment | 15 | We applied a fixed-effect model to assess treatment effects. A p-value < 0.05 was considered statistically significant. Publication bias was illustrated using a funnel plot. | 4 |
| **RESULTS** | | |  |
| Study selection | 16a | The screening process is shown in Figure 1 | 4 |
|  | 16b | Duplicate publication,wrong randomization,wrong intervention,not relevant to outcome measures,incomplete data | 4 |
| Study characteristics | 17 | Described in the Study characteristics | 4 |
| Risk of bias in studies | 18 | Described in the Publication bias | 5 |
| Results of individual studies | 19 | Described in the Study characteristics | 4 |
| Results of syntheses | 20a | Quality assessment | 4 |
|  | 20b | Described in the results of recurrence rate, pregnancy rate, serum level of CA-125 and adverse events | 4 |
|  | 20c | None of the studies used allocation, and all studies showed a high risk of bias due to the lack of using a placebo in control group. All studies were unclear on the blinding of outcome assessment and the selective reporting outcome. Other biases in all studies were also assessed as unclear due to a shortage of sufficient information. | 4 |
|  | 20d | Described in the subgroup analyses and sensitivity analysis | 5 |
| Reporting biases | 21 | Described in the publication bias | 5 |
| Certainty of evidence | 22 | Described in the publication bias | 5 |
| **DISCUSSION** | | |  |
| Discussion | 23a | Described in the discussion. | 5 |
|  | 23b | Described in the discussion. | 5 |
|  | 23c | Described in the discussion. | 5-6 |
|  | 23d | Described in the discussion. | 6 |
| **OTHER INFORMATION** | | |  |
| Registration and protocol | 24a | All included studies were not registered in China. | 6 |
|  | 24b | Indicate where the review protocol can be accessed, or state that a protocol was not prepared. | 6 |
|  | 24c | Describe and explain any amendments to information provided at registration or in the protocol. | 6 |
| Support | 25 | Described in the Funding. | 6-7 |
| Competing interests | 26 | Declare any competing interests of review authors. | 7 |
| Availability of data, code and other materials | 27 | Report which of the following are publicly available and where they can be found: template data collection forms; data extracted from included studies; data used for all analyses; analytic code; any other materials used in the review. | 7 |

*From:*  Page MJ, McKenzie JE, Bossuyt PM, Boutron I, Hoffmann TC, Mulrow CD, et al. The PRISMA 2020 statement: an updated guideline for reporting systematic reviews. BMJ 2021;372:n71. doi: 10.1136/bmj.n71

For more information, visit: <http://www.prisma-statement.org/>
